# Embryo Culture Duration Is an Independent Risk Factor for Gestational Diabetes in Frozen Embryo Transfer Pregnancies

**DOI:** 10.1210/jendso/bvaf161

**Published:** 2025-10-17

**Authors:** Huijun Chen, Shujuan Ma, Yvonne Liu, Yifan Gu, Fei Gong, Philipp Kalk, Carl-Friedrich Hocher, Ge Lin, Berthold Hocher

**Affiliations:** Department of Nephrology, Charité Universitätsmedizin Berlin, 10117 Berlin, Germany; Clinical Research Center for Reproduction and Genetics in Hunan Province, Reproductive and Genetic Hospital of CITIC-Xiangya, 410013 Changsha, Hunan, China; Clinical Research Center for Reproduction and Genetics in Hunan Province, Reproductive and Genetic Hospital of CITIC-Xiangya, 410013 Changsha, Hunan, China; Department of Nephrology, Charité Universitätsmedizin Berlin, 10117 Berlin, Germany; Clinical Research Center for Reproduction and Genetics in Hunan Province, Reproductive and Genetic Hospital of CITIC-Xiangya, 410013 Changsha, Hunan, China; Clinical Research Center for Reproduction and Genetics in Hunan Province, Reproductive and Genetic Hospital of CITIC-Xiangya, 410013 Changsha, Hunan, China; Institute of Reproductive and Stem Cell Engineering, NHC Key Laboratory of Human Stem Cell and Reproductive Engineering, School of Basic Medical Science, Central South University, 410083 Changsha, Hunan, China; Key Laboratory of Stem Cells and Reproductive Engineering, Ministry of Health, 410000 Changsha, China; Clinical Research Center for Reproduction and Genetics in Hunan Province, Reproductive and Genetic Hospital of CITIC-Xiangya, 410013 Changsha, Hunan, China; Institute of Reproductive and Stem Cell Engineering, NHC Key Laboratory of Human Stem Cell and Reproductive Engineering, School of Basic Medical Science, Central South University, 410083 Changsha, Hunan, China; Key Laboratory of Stem Cells and Reproductive Engineering, Ministry of Health, 410000 Changsha, China; Department of Nephrology, Charité Universitätsmedizin Berlin, 10117 Berlin, Germany; Diaverum Renal Care Center, Diaverum MVZ Am Neuen Garten Standort Ludwigsfelde, 14974 Potsdam, Germany; Fifth Department of Medicine (Nephrology/Endocrinology/Rheumatology/Pneumology), University Medical Centre Mannheim, University of Heidelberg, 68167 Mannheim, Germany; Clinical Research Center for Reproduction and Genetics in Hunan Province, Reproductive and Genetic Hospital of CITIC-Xiangya, 410013 Changsha, Hunan, China; Institute of Reproductive and Stem Cell Engineering, NHC Key Laboratory of Human Stem Cell and Reproductive Engineering, School of Basic Medical Science, Central South University, 410083 Changsha, Hunan, China; Key Laboratory of Stem Cells and Reproductive Engineering, Ministry of Health, 410000 Changsha, China; Clinical Research Center for Reproduction and Genetics in Hunan Province, Reproductive and Genetic Hospital of CITIC-Xiangya, 410013 Changsha, Hunan, China; Institute of Reproductive and Stem Cell Engineering, NHC Key Laboratory of Human Stem Cell and Reproductive Engineering, School of Basic Medical Science, Central South University, 410083 Changsha, Hunan, China; Fifth Department of Medicine (Nephrology/Endocrinology/Rheumatology/Pneumology), University Medical Centre Mannheim, University of Heidelberg, 68167 Mannheim, Germany; Hunan International Scientific and Technological Cooperation Base of Development and Carcinogenesis, 410000 Changsha, China; Institute for Medical Diagnostics Berlin (IMD Berlin), 12247 Berlin, Germany

## Abstract

**Objective:**

To investigate whether prolonged embryo culture increases the risk of gestational diabetes mellitus (GDM) in pregnancies conceived via frozen embryo transfer (FET).

**Research Design and Methods:**

In this retrospective cohort study, 26 100 FET pregnancies from 2018 to 2022 were analyzed. GDM was diagnosed by a 75-g oral glucose tolerance test. Embryo culture duration (day 3 vs day 5 vs day 6) and morphology were evaluated. Multivariable logistic regression adjusted for maternal age, body mass index, and fasting glucose. Interaction analyses assessed the combined effect of blastocyst transfer and maternal metabolic risk factors.

**Results:**

GDM occurred in 14.0% of pregnancies. Blastocyst-stage transfers were associated with a significantly higher GDM risk than cleavage-stage transfers (day 3: 15.1%; day 5: 17.4%; day 6: 18.2%; *P* = .01). Prolonged embryo culture remained an independent risk factor in adjusted models (odds ratio, 1.045; 95% CI, 1.019-1.071; *P* < .01). Embryo morphology showed no significant association with GDM. The combination of blastocyst transfer and maternal metabolic risk factors further increased GDM incidence.

**Conclusion:**

Prolonged embryo culture is an independent risk factor for GDM after FET. These findings suggest embryo development stage influences maternal glucose metabolism and should be considered in assisted reproductive technology protocols, particularly for women with existing metabolic vulnerabilities.

Gestational diabetes mellitus (GDM) is a significant pregnancy complication characterized by glucose intolerance with onset or first recognition during pregnancy [1]. Its prevalence has been rising globally, posing considerable challenges to maternal and fetal health [1]. Among women undergoing in vitro fertilization (IVF), GDM has garnered particular attention due to potential associations with the use of assisted reproductive technologies (ART) and with underlying patient characteristics.

The advent of IVF has revolutionized infertility treatment, offering hope to millions of individuals and couples worldwide. However, pregnancies achieved through IVF differ from those conceived naturally in several aspects, including maternal demographics, obstetric outcomes, metabolic sequelae, and perinatal risks [2, 3]. Studies consistently show that women who conceive through IVF are at a higher risk of developing GDM compared to those who conceive naturally [4-8]. Notably, in terms of the fertilization method, a higher risk of GDM was observed after ART compared to spontaneous conception in pregnancies following IVF, but not after intracytoplasmic sperm injection (IVF: risk ratio [RR], 1.95; 95% CI, 1.56-2.44; intracytoplasmic sperm injection: RR, 1.42; 95% CI, 0.94-2.15). Additionally, regarding the type of embryo transfer (ET), a higher risk of GDM after ART vs spontaneous conception was identified in fresh ET but not in frozen ET (FET) (fresh ET: RR, 1.38; 95% CI, 1.03-1.85; FET: RR, 0.46; 95% CI, 0.10-2.19) [2].

More specifically, in fresh ET cycles, women who underwent blastocyst transfer exhibited a significantly higher risk for GDM compared to those who underwent cleavage embryo transfer (21.15% vs 14.85%, *P* = .009; odds ratio [OR], 1.56; 95% CI, 1.12-2.18), as demonstrated in our previous cohort study [9]. This result provided novel insight into ART procedure-related risk factors for GDM and raises important questions about whether embryo-related factors, such as culture duration (eg, day [d] 3, d5, or d6 embryos) or morphology, contribute to GDM risk. However, prior studies addressing this issue have been limited by small sample sizes or focused on fresh ET cycles, leaving uncertainty about the relationship between these factors and GDM in FET cycles.

To address these gaps, larger studies are urgently needed to clarify the role of embryo culture duration and morphology in influencing GDM risk. This study aims to investigate these associations in a large cohort of women undergoing FET cycles.

## Materials and Methods

### Participants

The specific inclusion and exclusion criteria were outlined as follows:

Inclusion criteria:

1. women who received FET cycles
2. age between 18 and 45 years
3. nature cycle or artificial cycle was employed for endometrium preparation

Exclusion criteria:

1. uterine malformations (uterine septum ≥0.6 cm [identified by hysteroscopy or 4-dimensional color Doppler ultrasound], single-horned uterus, double uterus)
2. down-regulation artificial cycles
3. untreated hydrosalpinx

We excluded women with uterine malformations, down-regulation artificial cycles, or untreated hydrosalpinx to minimize confounding. Uterine malformations and untreated hydrosalpinx are associated with impaired implantation and adverse outcomes, whereas variations in endometrial preparation protocols could introduce heterogeneity in implantation and placentation processes. These exclusions ensured a more homogeneous cohort for evaluating the impact of embryo culture duration on GDM risk.

4. prediagnosed diabetes.

For participants with regular menstruation and ovulation, a natural cycle protocol was used for endometrial preparation. Continuous transvaginal ultrasound monitoring was performed from d8 to d12 of the menstrual cycle to track ovulation. Progesterone was administered for luteal phase support after ovulation. Cleavage-stage embryos were transferred 3 days after ovulation, whereas blastocysts were transferred 5 days after ovulation. If pregnancy was achieved, luteal phase support was continued until 10 weeks’ gestation. For women with irregular menstruation or ovulation, artificial cycles were employed by using exogenous hormones to prepare the endometrium for embryo transfer. Participants began daily administration of 4 to 8 mg of exogenous estrogen on the third day of their menstrual cycle to stimulate endometrial growth over approximately 10 to 14 days, with ultrasound monitoring to assess endometrial thickness. When the endometrium reached a thickness of at least 7 mm, progesterone was introduced to induce endometrial transformation. Cleavage-stage embryos were transferred 3 days after the initiation of progesterone, whereas blastocysts were transferred 5 days following progesterone administration. If pregnancy was achieved, exogenous estrogen was continued until 8 weeks’ gestation, and progesterone was continued until 10 weeks’ gestation.

### Outcome Measurement

Participants achieving pregnancy underwent GDM screening using an oral glucose load. Specifically, a 75-g oral glucose tolerance test (OGTT) was administered in line with established guidelines at approximately 20 weeks of gestation or later [10]. Pregnant women adhered to a normal diet for 3 days before fasting for at least 8 hours before the OGTT. During the test, they consumed 75 g of glucose dissolved in 300 mL of water within 5 minutes. Venous blood samples were taken at baseline (fasting) and at 1 and 2 hours following glucose intake. Blood glucose levels were measured using the glucose oxidase method. The diagnostic thresholds for GDM based on the 75-g OGTT were as follows: a fasting blood glucose level of ≥5.1 mmol/L (92 mg/dL), a 1-hour level of ≥10.0 mmol/L (180 mg/dL), and a 2-hour level of ≥8.5 mmol/L (153 mg/dL) [10].

### Follow up

Follow-up information on pregnancy complications and perinatal outcomes was obtained retrospectively from hospital medical records where the women gave birth. When necessary, additional information was confirmed by telephone contact with the patients.

### Statistical Analysis

Primary data analysis was conducted using the Statistical Package for the Social Sciences (SPSS), version 29.0 (SPSS Inc., Chicago, IL, USA). Homogeneity of variance and data normality were evaluated with the Levene test and the Kolmogorov-Smirnov test, respectively. Descriptive statistics were reported as frequency (%) or median with interquartile range, whereas continuous data were presented as mean ± SD. Categorical variables were analyzed using the χ² test, and continuous variables were assessed with the Mann-Whitney *U* test. To control for confounding factors, a multivariate regression model was developed. Graphs were created using GraphPad Prism 8 (GraphPad Software, San Diego, CA, USA). Forest plots were generated using RStudio 2024 (Posit PBC, Boston, MA, USA). Stratified and interaction analyses were performed to clarify the impact of embryo state and baseline risk factors. Statistical significance was set at *P* < .05.

### Approval

This retrospective cohort study was conducted from January 2018 to December 2022. It received approval from the Ethics Committee of the Reproductive and Genetic Hospital of CITIC-Xiangya (approval number: LL-SC-2024-028). Written informed consent was not required, as this study involved retrospective data analysis.

## Results

### Study Population and Baseline Characteristics

**Figure 1.**
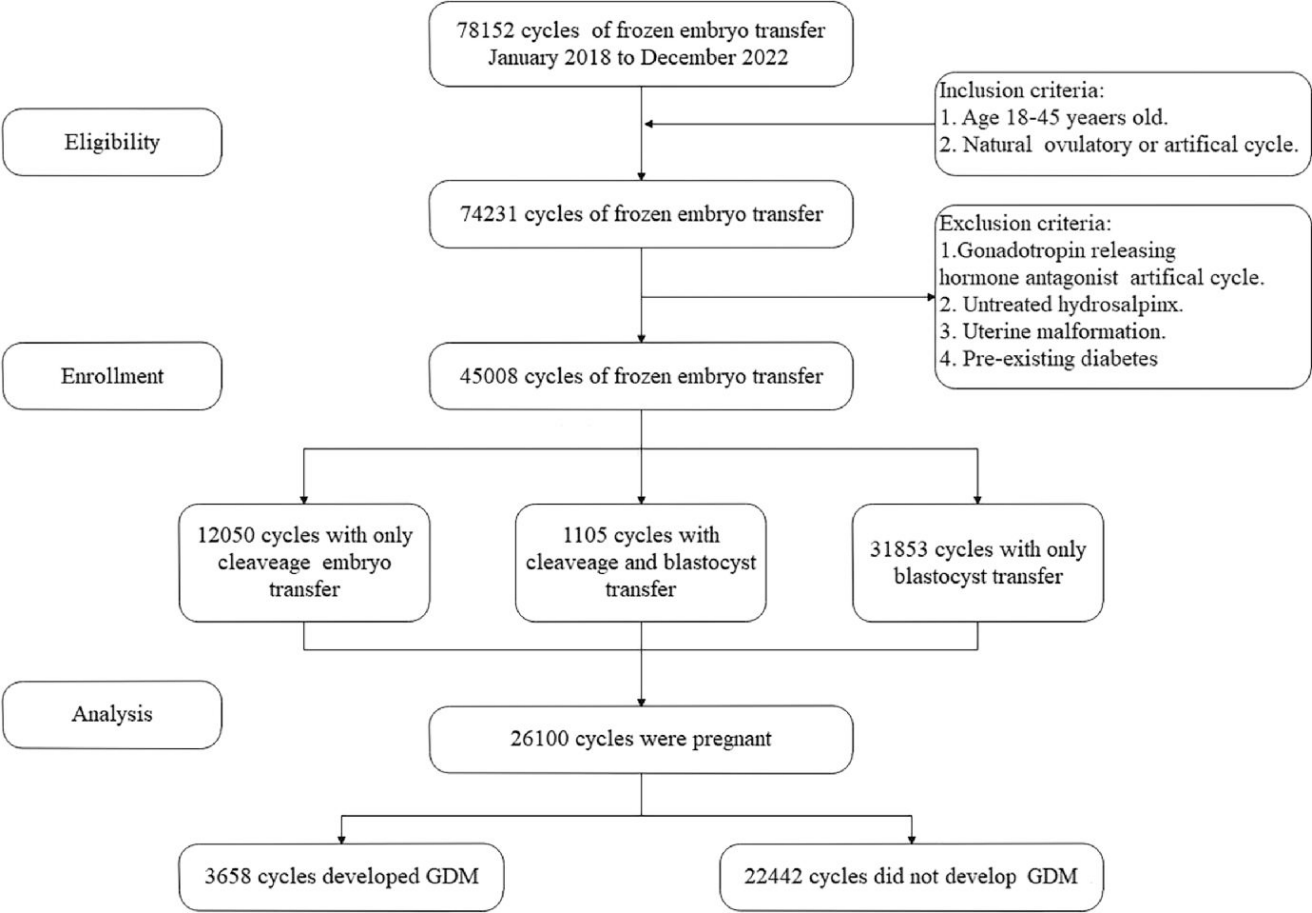
Study flow chart.

A total of 78 152 FET cycles were initially screened for eligibility. Cases were excluded based on the following criteria: (1) missing data (n = 3921), (2) uterine malformation or untreated hydrosalpinx (n = 5196), (3) preexisting diabetes mellitus (n = 174), and (4) down-regulation artificial cycles (n = 23 853). After applying these exclusion criteria, 45 008 cycles were eligible for our study and 26 100 pregnancies were included for the final analysis (Fig. 1). The overall pregnancy rate following FET was 57.99% (26 100/45 008).

The overall incidence of GDM in the cohort was 14.0% (3658/26 100). Baseline characteristics revealed significant differences between women who developed GDM and those who did not. Women with GDM had higher maternal age (*P* < .01), body mass index (BMI) (*P* < .01), waist-to-hip ratio (*P* < .01), and fasting glucose levels (*P* < .01). Additionally, the incidence of polycystic ovary syndrome (PCOS) (7.85% vs 6.66%, *P* = .01) and intrauterine adhesions (24.25% vs 22.31%, *P* = .01) was higher in the GDM group. Details of the study population are given in Table 1.

**Table 1.** The demographic information of participants

	All (n = 26 100)	GDM (n = 3658)	Non-GDM (n = 22 442)	*P* value
Age (year)	32.00 (29.00, 35.00)	33.00 (30.00, 36.00)	32.00 (29.00, 35.00)	<.01
Infertility type (%)				
Primary	24.61 (6424/26 100)	23.70 (867/3658)	24.76 (5557/22 442)	
Secondary	66.59 (17 381/26 100)	67.14 (2456/3658)	66.50 (14 925/22442)	.32
Others	8.79 (2295/26 100)	9.16 (335/3658)	8.73 (1960/22 442)	
BMI (kg/m^2^)	21.62 (19.98, 23.23)	22.03 (20.44, 23.52)	21.48 (19.91, 23.14)	<.01
Waist-to-hip ratio	0.80 (0.77, 0.84)	0.81 (0.77, 0.85)	0.80 (0.76, 0.84)	<.01
Infertility duration (year)	3.00 (2.00, 5.00)	3.00 (1.00, 5.00)	3.00 (2.00, 5.00)	.33
Fasting glucose (mmol/L)	5.05 (4.72, 5.34)	5.17 (4.82, 5.49)	5.03 (4.71, 5.31)	<.01
Embryo transferred (%)				
Cleavage embryo	24.75 (6460/26 100)	21.98 (804/3658)	25.20 (5656/22 442)	<.01
Blastocyst	75.25 (19 640/26 100)	78.02 (2854/3658)	74.80 (16 786/22 442)	
The number of embryos*^a^* transferred	1.47 ± 0.50	1.45 ± 0.50	1.48 ± 0.50	.01
Cleavage embryo*^a^*	0.51 ± 0.85	0.46 ± 0.82	0.51 ± 0.85	.01
Blastocyst*^a^*	0.97 ± 0.69	1.00 ± 0.67	0.97 ± 0.69	.01
The number of good-quality embryos transferred*^a^*	0.98 ± 0.71	0.96 ± 0.69	0.98 ± 0.71	.06
Cleavage embryo*^a^*	0.48 ± 0.83	0.44 ± 0.80	0.49 ± 0.83	<.01
Blastocyst*^a^*	0.50 ± 0.53	0.52 ± 0.53	0.49 ± 0.53	.01
PGT embryo transferred	9.67 (2523/26 100)	9.21 (337/3658)	9.74 (2186/22 442)	.32
Endometrial preparation (%)				
Natural cycles	90.70 (23 674/26 100)	89.56 (3276/3658)	90.89 (20 398/22 442)	.01
Artificial cycles	9.30 (2426/26 100)	10.44 (382/3658)	9.11 (2044/22 442)	
Endometrial thickness (mm)	11.70 (10.50, 12.90)	11.60 (10.50, 12.90)	11.70 (10.50, 12.90)	.07
Previous diseases (%)				
Hypertension	0.36 (94/26 100)	0.25 (9/3658)	0.38 (85/22 442)	.21
PCOS	6.83 (1782/26 100)	7.85 (287/3658)	6.66 (1495/22 442)	.01
Intrauterine adhesion	22.58 (5893/26 100)	24.25 (887/3658)	22.31 (5006/22 442)	.01
Endometriosis	5.22 (1362/26 100)	5.63 (206/3658)	5.15 (1156/22 442)	.23

Abbreviations: BMI, body mass index; GDM, gestational diabetes mellitus; PCOS, polycystic ovarian syndrome; PGT, peri-implantation genetic testing.

^a^Data are not normally distributed but presented as mean ± SD.

### Association Between Embryo Culture Duration and GDM Risk

**Figure 2.**
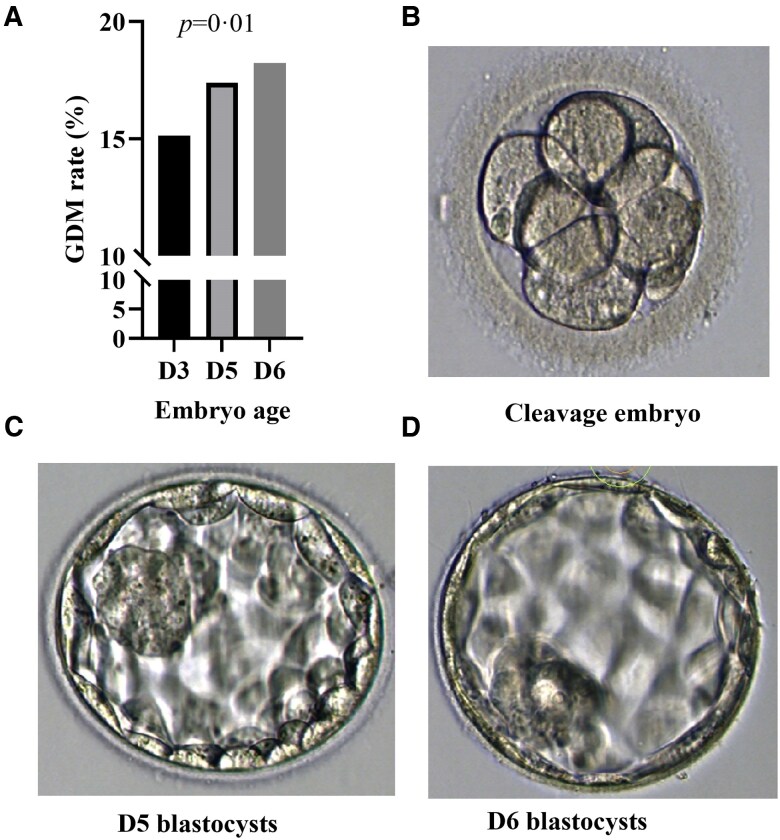
Gestational diabetes mellitus (GDM) rate and embryos.

Extended embryo culture duration was significantly associated with an increased risk of GDM. The incidence of GDM was highest among women who received d6 blastocysts transfer (d3: 15.13% [815/5387] vs d5: 17.39% [588/3381] vs d6: 18.24% [108/592], *P* = .01) (Fig. 2A-2D). Multivariable logistic regression confirmed this association after adjusting for maternal age, BMI, waist-to-hip ratio, and fasting glucose levels (OR, 1.045; 95% CI, 1.019-1.071; *P* < .01) (Table 2).

**Table 2.** Multivariate regression analysis to adjust the embryo stage on the incidence of GDM

	OR	95% CI		*P* value
Age	0.966	0.952	0.979	<.01
Waist-to-hip ratio	0.999	0.557	1.792	>.99
BMI	1.096	1.069	1.124	<.01
Fasting glucose	1.031	0.993	1.071	.12
PCOS	0.847	0.686	1.045	.12
Intrauterine adhesion	0.843	0.725	0.979	.03
Embryo stage (d3, d5, d6)	1.045	1.019	1.071	<.01

Abbreviations: BMI, body mass index; d, day; OR, odds ratio; PCOS, polycystic ovarian syndrome.

In this model, embryo development after fertilization was analyzed in days, as a continuous parameter. Confounding factors were identified using univariate regression analysis of the baseline parameters that exhibited significant differences (Table S5) and literature review.

### Comparison Between Cleavage Embryo and Blastocyst Transfer

A direct comparison between cleavage embryo and blastocyst transfer revealed significant differences in GDM incidence. Women who underwent blastocyst transfers had a higher risk of developing GDM compared to those who received cleavage-stage embryos (OR, 1.114; 95% CI, 1.011-1.229; *P* = .03) (Table S1) [11]. However, the number of embryos transferred was not related to GDM (Table S2) [11].

### Impact of Embryo Morphology on GDM Risk

No significant association was observed between the cell number of cleavage-stage embryos and GDM risk (OR, 1.042; 95% CI, 0.918-1.184; *P* = .52) (Table S3) [11].

Morphological parameters, including the blastocyst expansion stage (OR, 1.034; 95% CI, 0.974-1.097; *P* = .27), inner cell mass (ICM) grade (OR, 1.078; 95% CI, 0.952-1.220; *P* = .24), and trophectoderm (TE) grade (OR, 0.965; 95% CI, 0.896-1.039; *P* = .34) were also not significantly related to GDM incidence (Table S4A-C) [11]. Moreover, blastocyst quality (combining the expansion stage, ICM, and TE together), graded as excellent, good, average, or poor, also showed no significant association with GDM (OR, 1.009; 95% CI, 0.956-1.065; *P* = .74) (Table S4D) [11]. The distribution of blastocysts in single-embryo transferred participants is shown in Table 3.

**Table 3.** Distribution of composite morphology parameters of transferred blastocysts (single embryo transferred)

EH stage	n					ICM and TE grades				
		AA	AB	AC	BA	BB	BC	CA	CB	CC
6	3256 (15.39)	524 (15.84)	433 (12.24)	23 (13.04)	306 (11.76)	1455 (16.29)	497 (17.30)	4 (0)	14 (21.43)	—
5	2309 (14.42)	93 (23.66)	305 (11.15)	19 (15.79)	111 (13.51)	1155 (13.94)	620 (15.81)	—	6 (0)	—
4	12 049 (12.32)	382 (13.09)	1036 (13.80)	294 (12.24)	187 (16.04)	3744 (15.20)	6362 (14.02)	2 (0)	41 (14.29)	—
3	56 (16.07)	—	3 (0)	—	—	11 (18.18)	42 (16.67)	—	—	—
2*^a^*	168 (17.26)	—	—	—	—	—	—	—	—	—
1*^a^*	65 (13.63)	—	—	—	—	—	—	—	—	—
Total	17 903 (14.56)	999 (15.52)	1777 (12.94)	336 (12.50)	604 (13.41)	6365 (15.22)	7521 (14.40)	6 (0)	62 (14.52)	—

Abbreviations: EH, expanding and hatching; ICM, inner cell mass; TE, trophectoderm.

Values are n (GDM rate, %).

^a^ICM and TE grades were not evaluated.

### Stratified Analysis

**Figure 3.**
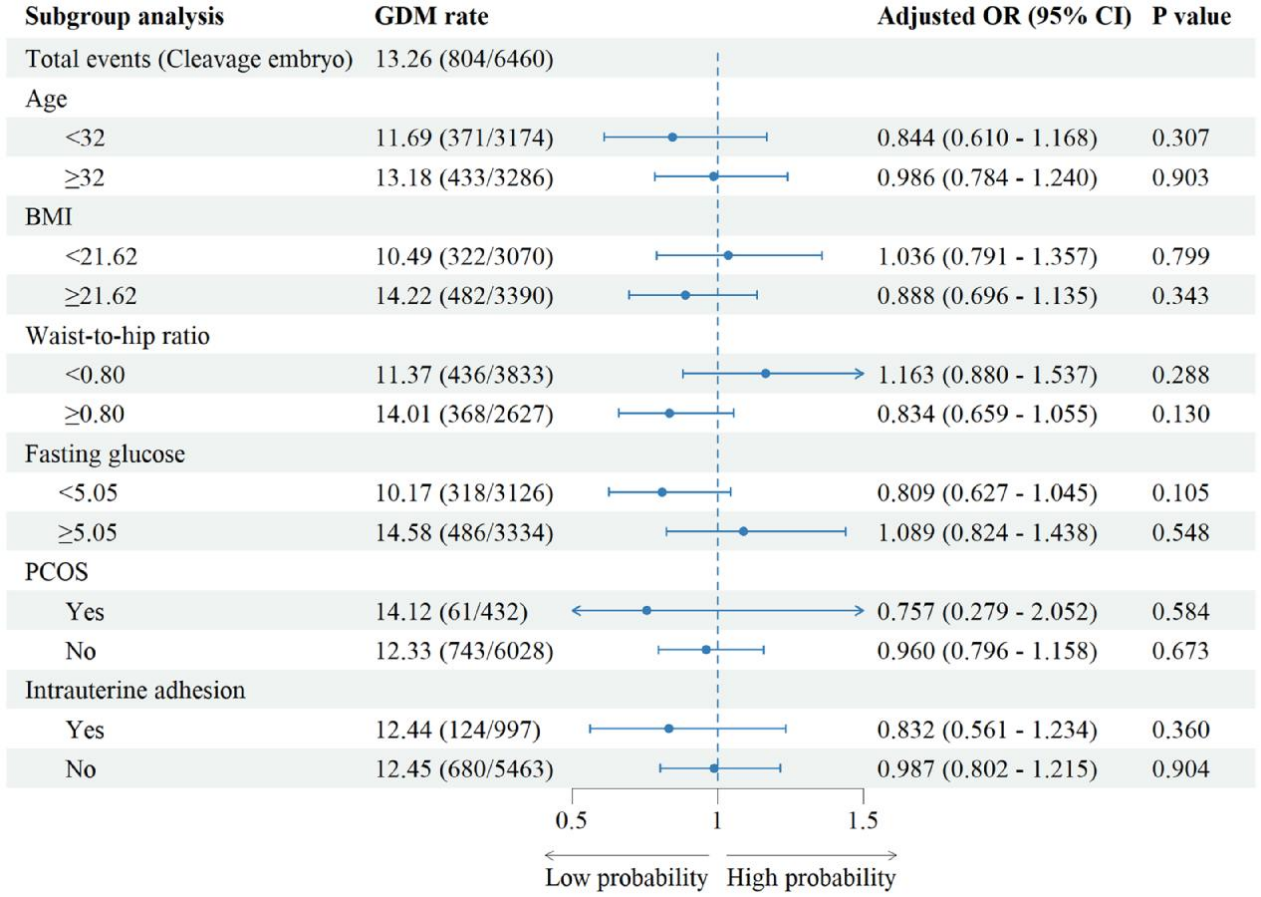
Stratified analysis of the whole population to investigate the impact of blastocyst transfer vs cleavage embryo transfer on GDM rate.

A stratified analysis was conducted to assess and optimize the incidence of GDM within the population. Participants were divided into 2 groups based on median values of key factors. The results revealed that women aged >32 years (*P* = .01), with a BMI exceeding 21.62 kg/m² (*P* = .05), a waist-to-hip ratio >0.80 (*P* = .04), fasting glucose levels >5.05 mmol/L (*P* = .01), or with intrauterine adhesions (*P* < .01) exhibited a higher incidence of GDM when transferring blastocysts than cleavage embryos (Fig. 3, Table S5) [11].

Further stratified analyses were performed to determine the impact of embryo morphology on GDM incidence among women who transferred either cleavage-stage embryos or blastocysts. The results indicated that the grade of cleavage-stage embryos was not associated with GDM incidence across different populations (Fig. S1) [11]. However, in women aged >32 years, blastocyst grade was associated with an increased incidence of GDM (OR, 1.092; 95% CI, 1.017-1.171; *P* = .02) (Fig. S2) [11].

### Interaction of Embryo State and Maternal Risk Factors of GDM.

**Figure 4.**
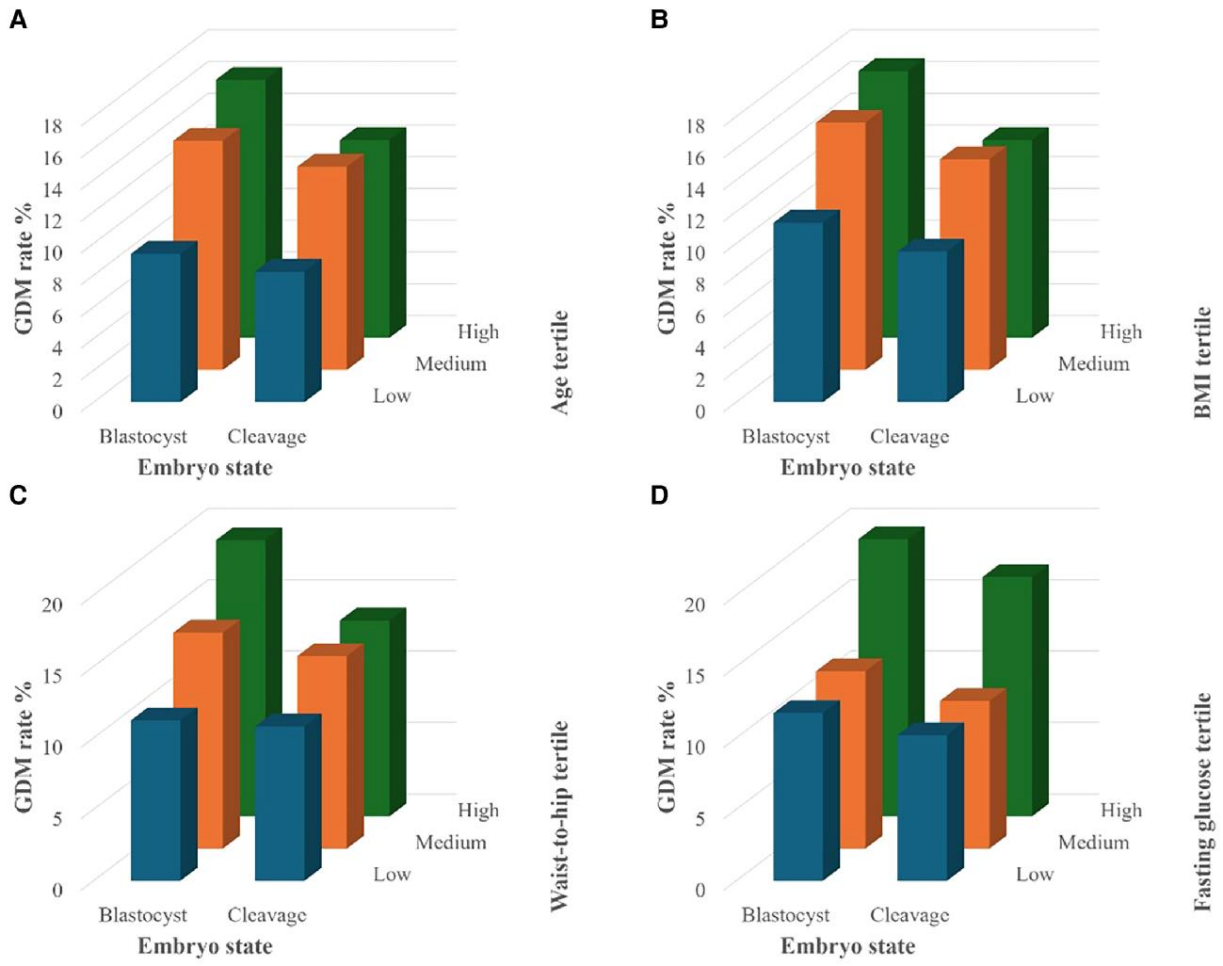
3D plots of the effects of embryo stage and baseline risk factors on GDM incidence.

Interaction analysis demonstrated that the combination of blastocyst transfer with maternal risk factors—such as higher BMI, higher waist-to-hip ratio, advanced maternal age, and elevated fasting glucose levels—significantly increased GDM incidence beyond the effect of individual risk factors (Fig. 4A-4D).

### Sensitivity Analysis

We performed the sensitivity analysis to further verify the stability of our regression models. In this analysis, we added the parameters with *P* value lower than either 0.1 (first set of models) or 0.2 (second set of models) instead of *P* < .05 as described previously from the demographic analysis to the previous regression models. Our findings remained stable regardless of the applied model (Table S6-S9) [11].

## Discussion

### Summary of Key Findings and Implications

This study demonstrates that prolonged embryo culture is significantly associated with an increased risk of GDM in pregnancies conceived via FET. Although embryo morphology did not independently influence GDM risk, our findings indicate that the timing of embryo culture—specifically blastocyst-stage transfer—interacts with maternal metabolic factors such as BMI and fasting glucose to further elevate risk. These results suggest that embryo culture duration is not a neutral parameter and may have important implications for maternal metabolic health. Reevaluating embryo culture practices within ART protocols may help reduce GDM incidence. Integrating embryo developmental stage into individualized ART planning, particularly for women with preexisting metabolic vulnerabilities, could improve pregnancy outcomes and support safer fertility treatment strategies.

### Days of Embryo Culture and GDM Risk

Our study revealed that transferring embryos at the blastocyst stage (d5 or d6) significantly increases GDM risk compared to cleavage-stage transfers (d3). This aligns with findings from previous human studies, which suggest that extended in vitro culture may alter embryo-endometrial synchronization, affecting placental development and maternal metabolic adaptations.

Recent human studies have suggested that extended embryo culture to the blastocyst stage may lead to increased implantation rates but also higher risks of pregnancy complications, including hypertensive disorders and metabolic changes [12-14].

Slower blastocyst development may be caused by delayed timing of the first cleavage division, and subsequent cleavage divisions, which may influence the developmental timeline of the embryo [15]. Such slow development might impair the biochemical, metabolic, and epigenetic processes of blastocysts [16]. Animal studies suggest that a delay in blastocyst formation or an additional day of culture could increase the incidence of apoptosis, decrease the ICM:TE cell ratio, and influence the gene expression and diameter of blastocysts derived from in vitro-produced pig embryos [17]. These findings indicate that impaired blastocyst quality may influence fetal programming and maternal glucose metabolism, warranting further investigation.

### Comparison of Classical GDM Risk Factors in Cleavage Embryo Versus Blastocyst Transfer

One critical factor in interpreting our findings is whether maternal risk factors for GDM, such as BMI, waist-to-hip ratio, age, and baseline glucose metabolism, differed among the d3, d5, and d6 embryo transfer groups. In our cohort, maternal characteristics, including age and BMI, were comparable between groups. The fasting glucose was even lower in the d6 embryo transfer group (Table S5) [11]. However, subtle differences in preexisting metabolic risk factors could potentially contribute to the observed association between extended embryo culture and increased GDM risk. Our study also reported that women undergoing blastocyst transfer may have higher baseline fertility-related risks, such as PCOS, which are known to increase GDM risk [18, 19]. Nonetheless, our regression models adjusted for these confounders, suggesting that the observed association between embryo transfer timing and GDM risk is likely independent of classical risk factors. Future studies should explore whether additional metabolic biomarkers, such as insulin resistance indices or lipid profiles, play a role in modifying this risk [20].

### Embryo Morphology and Pregnancy Metabolic Outcomes

Despite the significant association between embryo culture duration and GDM, our study did not find a direct link between embryo morphology (cleavage-stage cell number, blastocyst expansion, ICM quality, and TE grade) and GDM risk. Previous human studies have yielded mixed results regarding the impact of embryo morphology on pregnancy complications, with some suggesting that poor-quality embryos may have altered metabolic programming [21], whereas others report no significant effects [22-24].

Studies in animals have shown that poor-quality embryos exhibit altered epigenetic modifications, leading to changes in fetal growth and glucose homeostasis [17]. However, these effects appear to be more pronounced in fresh embryo transfers rather than frozen cycles [2], suggesting that cryopreservation may mitigate some of the developmental consequences of suboptimal embryo morphology. Given these findings, further studies are required to determine whether specific morphological features of blastocysts influence long-term metabolic health in ART-conceived pregnancies.

### Interaction Between Classical GDM Risk Factors and Embryo Culture Days as a Novel Risk Factor

We thank the reviewer for raising this important point. Indeed, baseline metabolic risk factors such as BMI, fasting glucose, and PCOS are well-established determinants of GDM. In our cohort, we observed subtle differences (eg, lower fasting glucose in the d6 embryo transfer group) (Table S5) [11]. However, these variations were carefully addressed in our multivariable regression models, which included adjustments for maternal BMI, age, waist-to-hip ratio, fasting glucose, and PCOS.

Importantly, our interaction analyses demonstrated that prolonged embryo culture increased GDM risk disproportionately in women with higher BMI or elevated fasting glucose, indicating a synergistic rather than purely confounding effect. Furthermore, the association between extended embryo culture and GDM risk remained statistically significant after adjustment for all classical risk factors, supporting the conclusion that embryo culture duration is an independent risk factor.

Although subtle differences in baseline metabolic profiles were present (eg, slightly lower fasting glucose in the d6 group), these were fully accounted for in our multivariable regression models. Notably, interaction analyses suggested a synergistic effect, with extended embryo culture conferring a disproportionate GDM risk in women with higher BMI or elevated fasting glucose. This supports the interpretation that embryo culture duration is an independent contributor to GDM risk, beyond the influence of classical metabolic factors such as BMI, glucose, or PCOS.

This suggests that extended embryo culture may exacerbate preexisting metabolic vulnerabilities, amplifying the risk of GDM beyond the impact of individual risk factors alone.

Existing literature on the relationship between maternal metabolic health and embryo culture days remains limited. However, studies have suggested that maternal glucose metabolism influences endometrial receptivity and embryo implantation success [25-27]. Current evidence, based on both human and animal models, has demonstrated that hyperglycemic conditions at the time of implantation can lead to aberrant placentation and fetal overgrowth, further supporting a link between metabolic status and embryo transfer outcomes [28, 29]. Future research should investigate whether specific metabolic interventions before FET, such as glucose-lowering treatments or dietary modifications, could mitigate the increased risk associated with blastocyst transfers. Our findings are consistent with recent evidence linking embryo culture duration and embryo developmental stage to perinatal outcomes in FET cycles. A large cohort study reported that prolonged embryo culture was associated with an increased risk of large-for-gestational-age infants in cryopreserved embryo transfer cycles [29]. Similarly, a systematic review and meta-analysis highlighted the influence of embryo stage on obstetric complications and perinatal outcomes, underscoring the importance of embryo developmental timing in both programmed and natural FET cycles [30]. Together with our results, these studies further support the concept that embryo culture conditions and stage at transfer represent critical factors shaping maternal and neonatal outcomes.

### Study Limitations

Despite the strengths of this study, including a large cohort and comprehensive adjustment for potential confounders, several limitations must be considered. First, as a retrospective observational study, this study’s causality cannot be established between embryo culture duration and GDM risk. Second, potential confounders such as dietary habits, physical activity, and genetic predisposition to metabolic disorders were not fully accounted for because they were not recorded, which may influence the observed associations. Third, embryo selection bias may exist because embryos that reach the blastocyst stage may inherently differ in developmental potential from those transferred at the cleavage stage. Last, although we adjusted for maternal baseline characteristics, future studies should incorporate molecular and epigenetic analyses to explore the underlying biological mechanisms of these associations. Also, we did not include a control group of women with spontaneous pregnancies and metabolic risk factors such as overweight, insulin resistance, or PCOS. Because our database covers only FET cycles, such a comparison was not possible. We accounted for these conditions by adjusting for BMI, fasting glucose, waist-to-hip ratio, and PCOS in our models. Importantly, embryo culture duration remained an independent predictor of GDM. Future studies combining ART and non-ART cohorts are needed to confirm and extend these findings. Although fasting glucose was included as a baseline metabolic marker, we did not have data on insulin levels or homeostatic model assessment for insulin resistance because these parameters are currently not part of the routine laboratory evaluation in our center, which limits our ability to fully assess insulin resistance. This is particularly relevant in PCOS, a heterogeneous syndrome in which ∼70% of women present with insulin resistance. In our dataset, PCOS was coded as a binary variable without detailed phenotyping. Thus, although PCOS was not independently associated with GDM after adjustment for BMI and fasting glucose, it remains possible that specific insulin-resistant PCOS subgroups contribute disproportionately to GDM risk. Future studies incorporating detailed metabolic profiling and PCOS phenotyping are needed to further clarify this relationship.

## Conclusions

This large retrospective cohort study demonstrates that prolonged embryo culture is an independent risk factor for GDM in pregnancies conceived through FET. Transfers at the blastocyst stage (d5 or d6) were associated with a significantly higher GDM incidence compared to cleavage-stage (d3) transfers, even after adjusting for maternal age, BMI, and fasting glucose levels. In contrast, embryo morphology was not associated with GDM risk.

Our findings suggest that in vitro culture duration may influence maternal metabolic outcomes, possibly by affecting embryo-endometrial synchrony, placental development, or early fetal programming. Notably, the risk of GDM was amplified when blastocyst transfer was combined with maternal metabolic risk factors such as elevated BMI or fasting glucose. These results underscore the importance of considering both embryonic and maternal characteristics when designing ART protocols.

Clinically, this study supports a more individualized approach to embryo transfer decisions. For women with preexisting metabolic vulnerabilities, cleavage-stage embryo transfer may reduce the risk of GDM without compromising overall pregnancy success. Furthermore, our results highlight a need to evaluate and optimize current embryo culture protocols to improve maternal-fetal health outcomes.

Although our data derive from a Chinese ART cohort with comparatively high background T2DM risk, the exposure of interest (embryo culture duration/stage at transfer) is not population-specific and remained associated with GDM after adjustment for BMI, fasting glucose, waist-to-hip ratio, and PCOS. Nevertheless, differences in metabolic profiles and clinical practice patterns across regions may influence the magnitude of risk, but not the type of risk. Future studies should validate these findings in ethnically diverse populations (Europe, North America, Australia) and in harmonized, multicenter datasets. Mechanistic research are warranted to clarify the biological pathways linking embryo culture conditions to maternal glucose metabolism. Ultimately, integrating embryological and metabolic considerations may enhance ART safety and long-term outcomes for both mothers and offspring.

## Data Availability

Some or all datasets generated and/or analyzed during the current study are not publicly available but are available from the corresponding author upon reasonable request.

## Abbreviations

ART: assisted reproductive technology
BMI: body mass index
d: day
ET: embryo transfer
FET: frozen embryo transfer
GDM: gestational diabetes mellitus
ICM: inner cell mass
IVF: in vitro fertilization
OGTT: oral glucose tolerance test
OR: odds ratio
PCOS: polycystic ovary syndrome
RR: risk ratio
TE: trophectoderm
